# Pelvic Reduction Frame Facilitating Percutaneous Fixation to Pelvic Fractures

**DOI:** 10.1111/os.13709

**Published:** 2023-03-14

**Authors:** Jeffrey Owen Anglen

**Affiliations:** ^1^ Orthopaedic Trauma, Alaska Native Medical Center Anchorage Alaska USA

## Abstract

This article is a retrospective report of the outcome of 43 unilateral unstable, vertically displaced pelvic ring injuries using a reduction frame which is attached to the OR table, along with special instruments allowing “unlocking” of the fracture to facilitate reduction. The frame utilized by the authors is a modification of the Starr Frame® (Starr Frame, LLC, Richardson), which has been refined to be lower profile, more accessible, and more versatile in pin placement. They have also reported a new attachment for the frame to apply lateral traction to the fractured hemipelvis. The authors provide a detailed description of the techniques involved in securing the patient to the frame and table, and then the stepwise application of multiplanar tractions to the fractured hemipelvis. Their technique of unlocking closed reduction (UCRT) involved controlled application of lateral distraction to disimpact the compressed or overlapping fractured bone surfaces, followed by distal and anterior traction through a femoral supracondylar pin, and direct manipulation of the hemipelvis through a supracetabular “LC‐2” half pin. Reduction is followed by percutaneous fixation using 7.3‐mm cannulated screws across the posterior ring and either external fixation or subcutaneous supra‐acetabular pedicle screw internal fixation device (INFIX) anteriorly.

We write this letter in response to this article entitled “Achieve Closed Reduction of Irreducible, Unilateral Vertically Displaced Pelvic Ring Disruption with an Unlocking Closed Reduction Technique” by Chen et al.^1^ published in *Orthopedic Surgery* (2021;13:942–948). Reduction of pelvic fractures with minimal traction will be the optimal approach. They developed a new pelvic reduction system based on unlocking closed reduction theory (UCRT). The UCRT proposed a new concept that decoupling bone fragments in the pelvic fracture is the key first step to allow reduction to be achieved by minimal traction. This involves stabilization of the intact hemipelvis and application of lateral traction, flexion/extension, and rotation through Schanz pins inserted into the ilium and connected to the frame. In addition, their team^2^ divided lateral compression type 2 Pelvic Fractures into two different kinds of fracture displacement: internal displacement only and a combination of internal, cephalic, and dorsal dislocation through the sacroiliac joint. Two different closed reduction strategies were suggested: the former one was first longitudinal traction and then transverse traction; the latter was first transverse traction then longitudinal and LC2 traction. Since 2014, they have led a national multi‐center UCRT study of pelvic fracture in China, which has treated 454 patients and achieved good results.^1^, ^2^, ^3^, ^4^ Tang and Chen are the pioneers in China, who have pushed the minimally invasive techniques of pelvic fracture forward in China. Much of the recent world literature on this topic has come from China. We congratulate the authors for their efforts.

Secondly, this article is a retrospective report of the outcome of 43 unilateral unstable, vertically displaced pelvic ring injuries using a reduction frame which is attached to the OR table, along with special instruments allowing “unlocking” of the fracture to facilitate reduction. The frame utilized by the authors is a modification of the Starr Frame® (Starr Frame, LLC, Richardson), which has been refined to be lower profile, more accessible, and more versatile in pin placement. They have also reported a new attachment for the frame to apply lateral traction to the fractured hemipelvis. Besides, many authors have paid much attention to the development of closed reduction tools. Matta et al.^5^ devised a table‐skeletal pelvic fixation frame to secure the normal side of the pelvis to the operating table. The goal of stabilization is to resist movement of the intact side of the pelvis as extrinsic reduction forces are applied. Sellei et al.^6^ designed an X‐frame with a standard single‐pin anterior supraacetabular external fixator that allows preloading of the Schanz pins, which was to improve the initial anterior and posterior stability for pelvis reduction manipulation. Queipo‐De‐Llano et al.^7^ used a pre‐tensed curved bar to reduce pelvic disruptions by applying compression simultaneously through the sacroiliac joint and the symphysis. Starr et al.^8^ developed a frame (The Starr Frame®, Starr Frame LLC, Richardson) comprised of two carbon‐fiber rings which are attached to the operating table and allow gradual realignment of the displaced hemipelvis and stable maintenance of reduction while fixation is placed. Lefaivre and Starr proposed two stabilization pins (lateral–medial supra‐acetabular pin and LC‐2 pin) to achieve multi‐planer fixation to stabilize the intact side of the pelvic ring, which established the operation basis of minimally invasive treatment of the pelvis. However, due to the circular structure of Starr frame, it cannot provide a flat area that clamps attach to, making the crossbar unstable and influencing reduction of dorsal or rotational displacement. In addition, the Starr frame is so large (width: 800 mm, height: 500 mm) that a regular 12‐inch image intensifier for fluoroscopy should be required for monitor of fracture displacement. However, in this study I did note that the average BMI in their patient population was 23.2 (range 18–31), which may limit the generalizability of their results to, for example, a North American population, where obesity is more prevalent. The principles of their technique should be generalizable to any patient population, and the modifications of the frame shape and design could certainly be upsized as needed.

Thirdly, the authors provide a detailed description of the techniques involved in securing the patient to the frame and table, and then the stepwise application of multiplanar tractions to the fractured hemipelvis. Their technique of unlocking closed reduction (UCRT) involved controlled application of lateral distraction to disimpact the compressed or overlapping fractured bone surfaces, followed by distal and anterior traction through a femoral supracondylar pin, and direct manipulation of the hemipelvis through a supracetabular “LC‐2” half pin. Reduction is followed by percutaneous fixation using 7.3‐mm cannulated screws across the posterior ring and either external fixation or subcutaneous supra‐acetabular pedicle screw internal fixation device (INFIX) anteriorly. In fact, the three‐dimensional multidirectional displacements of pelvic ring fractures, involving both anterior and posterior structures, play a role in the difficulty of definitive treatment. The tools mentioned above can only provide a single‐dimensional reduction and reducing the complex displaced disruptions can be difficult. Tools with the function of holding the patient still, pushing, pulling, or rotating the injured side in the right direction, and placing fixation in a “moving target” with sometimes narrow safe corridors are still being developed. The key to closed reduction is to apply the traction in the desired direction to achieve a good reduction of the fracture. This may involve multiplanar translation as well as rotational forces. But sometimes traction forces applied by the Matta frame are too high, especially for the irreducible or old fracture of more than 10 days after injury. Although the frictional forces generated by the weight of the patient on the operating table provide some resistance to traction, the forces necessary to reduce the pelvic ring can be greater than this, resulting in the patient being pulled distally. Besides, the pelvis can rotate around the post in response to unilateral traction. Additionally, the post may aggravate anterior pelvic ring deformity with pressure against mobile ramus fractures and/or resist the closure of a dislocated pubic symphysis.

Lastly, the complexity and variability of pelvic anatomy, the deep location, and the relationship to vital structures, together with the multiplanar displacement of the injuries combine to make closed reduction of displaced fractures and dislocations challenging. Early attempts to control displacement of pelvic fractures were primarily directed at saving lives by decreasing hemorrhage, for example with the use of Medical Anti‐Shock Trousers (MAST), and then definitive treatment with prolonged traction. Both were unsatisfactory and led to a host of complications. The pioneers of percutaneous stabilization developed several tools and methods of reduction. External fixation with pins in the iliac crest was employed, but the pins were difficult to place, had a tenuous grip on the innominate bone and did not allow the correct force vectors required to control hemipelvis reduction. Pin site problems were common and limiting. Heini et al. designed a pelvic C‐clamp as an external fixator for unstable pelvic fracture reduction. This device was primarily designed to “close the book” and control or decrease pelvic volume to control internal hemorrhage during the resuscitation phase of care. It was for temporary use and not intended to afford any degree of anatomical reduction other than closing the volume of the pelvis. It soon became apparent that it was associated with several major intraoperative complications related to the superior gluteal artery and nerve.^9^ Anterior external fixation was modified to place pins in the supra‐acetabular region from anterior inferior iliac spine (AIIS) to the posterior superior iliac spine (PSIS), which is known as the Hannover technique.^10^ The use of anterior external fixation does not control the posterior ring well and can even lead to worsening of posterior displacement. Notwithstanding these attempts to optimize external fixation, today emergent exfix has been supplanted using trochanteric binders, and the utilization of external fixation for definitive anterior ring control is mostly restricted to patients who cannot have internal fixation for some reason.

Conclusively, percutaneous reduction and fixation of pelvic fractures are the trends in treatment and the indications for its use are not yet fully defined. It is hoped that most orthopaedic surgeons can think about medicine using modular thinking, understand science and technology with medical logic, and embrace development with an integrated attitude. Minimally invasive treatment of pelvic fracture will develop rapidly and should become part of every orthopaedic traumatologist's repertoire of techniques. We appreciate that Tang and Chen has provided us with a clinically meaningful study.
